# Assessing the Acceptability and Feasibility of Five Cycles of Seasonal Malaria Chemoprevention in Côte d’Ivoire

**DOI:** 10.3390/tropicalmed10010010

**Published:** 2024-12-26

**Authors:** Orphée Kangah, Issaka Zongo, Alassane Haro, William Yavo

**Affiliations:** 1 National Institute of Public Health, Abidjan BP V 47, Côte d’Ivoire; yavowilliam@yahoo.fr; 2 Institut de Recherche en Sciences de la Santé—Direction Régionale de l’Ouest (IRSS-DRO), Bobo-Dioulasso BP 2779, Burkina Faso; zongoissaka08@gmail.com (I.Z.); alassaneharo@hotmail.com (A.H.)

**Keywords:** seasonal malaria chemoprevention, acceptability, feasibility, north Côte d’Ivoire

## Abstract

Seasonal malaria chemoprevention (SMC) is a strategy recommended by the World Health Organization for children aged 3–59 months in the Sahel and sub-Sahel regions where malaria transmission is seasonal. In Côte d’Ivoire, malaria remains a high priority and accounts for the majority of consultations and deaths in children under five. The recent revision of the criteria for the introduction of seasonal malaria chemoprevention has made the north of Côte d’Ivoire, where malaria transmission is seasonal, eligible for the SMC. We conducted a pilot study in this part of the country to assess the acceptability and feasibility of five cycles of SMC in 1701 children. Seasonal malaria chemoprevention with sulfadoxine–pyrimethamine + amodiaquine (SP + AQ) was administered monthly to eligible children over five months. A qualitative approach and quantitative surveys were used to assess the strategy acceptability and feasibility in the study area. Overall, there was a positive perception, attitude, and adherence towards the seasonal malaria chemoprevention in this study area.

## 1. Introduction

Seasonal malaria chemoprevention (SMC) is an effective malaria control strategy implemented on a large scale in countries where most clinical malaria cases occur during a limited rainy season [1,2]. It is increasingly recognized that malaria control strategies need to be adapted to the local context to maximize its impact. SMC is an example of a strategy designed for the countries of the Sahel and sub-Sahel and other areas where at least 60% of the annual malaria burden occurs during the short rainy season. Eligibility for SMC is determined by the seasonality of malaria transmission and the age groups at risk of severe malaria. In these areas of high seasonality for malaria transmission, the burden of malaria and severe malaria remains high. The number of malaria cases was estimated at 241 million in 2020 compared to 227 million in 2019, an increase of nearly 14 million cases. In 2020, 69,000 more people died of malaria than in 2019 (627,000 vs. 558,000). Approximately 95% of malaria cases were recorded in the WHO African Region. Most of these countries of high burden are in the seasonal transmission settings (Burkina Faso, Cameroon, Ghana, Mali, Niger, Nigeria) [3]. Ivory Coast is close to sub-Saharan countries, and a study conducted in Korogho has reported similar seasonality of clinical malaria being observed in these countries [4]. The WHO has established that the evaluation of criteria evolves over time and space, and that malaria control programs must assess the relevance of SMC according to local malaria epidemiology and available funding [2].

A study conducted in the western part of Côte d’Ivoire between March 2020 and May 2021 in children under 7 years old revealed that the prevalence of malaria was 66.7% and 78.3% based on microscopy and rapid diagnosis tests, respectively. Once again, the prevalence was higher among older children (aged 3–6 years) (77.8%) than among the youngest children (aged 3 months to 2 years) (58.3%) [5]. We conducted a pilot study in 2021 there with the objective of assessing the acceptability and feasibility of five cycles of SMC with sulfadoxine–pyrimethamine + amodiaquine (SP + AQ).

## 2. Materials and Methods

### 2.1. Study Area, Period, and Duration

The study was carried out in the Dikodougou health district in the Korhogo region, north of Côte d’Ivoire. This area is located in the southern part of the Sahelian strip. The climate regime is semi-arid [4], and it is the driest region in the country. The vegetation consists of dry forests and savannahs. A recent survey in the region revealed a prevalence of *Plasmodium* spp. infection of 12.4%; asymptomatic malaria infection was significantly associated with age and rainy season [6]. The incidence of cases in Dikodougou during the rainy season from July to October 2019 was 62%. The study took place early in June 2021 and ended late in December 2021, and covered the period of high malaria transmission. Accounting for geographical accessibility, Dikodougou city, Kadioha, Poundia, Sonzorisso, and Diendron were included in the study.

### 2.2. Type of Study

We carried out an acceptability and a feasibility study. In the acceptability study, we assigned a qualitative value to participants’ appreciation of, the strategy, while in the feasibility one, we considered key elements such as key SMC administration personnel identification, ease of training, ease of drug administration, and overall implementation factors, including logistics, storage, and workload.

### 2.3. Overall Study Procedures 

Prior to implementing chemoprevention in the field, a series of activities were performed: community and leader sensitization, field worker recruitment and training, and eligible child enumeration. Next, chemoprevention of seasonal malaria was implemented, involving the delivery of SMC drugs to children in two age groups (3–11 months and 12–59 months). SMC treatments were administered at one-month intervals over five consecutive months (from July to November 2021), covering the period of high transmission. At the beginning of each month, community health workers visited families to administer the first dose of treatment to children, provide them with an SMC ID card, and leave the remaining doses with the mothers to administer on the second and third days. Dispersible SP and AQ tablets were used to facilitate administration. The treatment regimen for a child aged 12–59 months was a single course of SP (500 mg sulfadoxine/25 mg pyrimethamine) and AQ 150 mg on day one and AQ 150 mg on days two and three. Infants under one year of age received 250 mg sulfadoxine/12.5 mg pyrimethamine and AQ 75 mg on day 1 and AQ 75 mg on days 2 and 3.

### 2.4. Inclusion and Non-Inclusion Criteria

Children fulfilling the listed inclusion criteria were enrolled in the study: children aged at least 3 months and less than 60 months, of either sex, living in the study area with no plan to move during the study period, with an absence of severe or chronic disease (severe malnutrition, children with aids), and with no known allergies to AQ or SP. Finally, informed consent was provided by the participants’ parents or legal guardians. Children who did not fulfill the above criteria were not invited to participate. Children presenting with clinical malaria were enrolled but were treated on the subsequent cycle of SMC.

### 2.5. Informed Consent Procedure

A careful informed consent procedure was implemented by the investigators for each child, and only those whose parents agreed to participate in the study were enrolled. After verbal acceptance, individual written informed consent was obtained from the parent or legal guardian at enrolment for the study.

### 2.6. Individual Interview and Focus Group Discussions on Acceptability and Feasibility

Two structured questionnaires were individually administrated to the mothers or the children’s guardians in the families: the first questionnaire was designed to collect data on acceptability after the awareness campaign, and the second was used to assess acceptability in terms of the proportion of daily doses administered to the children by the mothers. Semi-structured interview guides were used for in-depth interviews with healthcare staff at various levels, and for focus groups with mothers and heads of household. The aim was to evaluate their acceptance of the strategy and how feasible it will be. The questions asked of the mothers and heads of household included knowledge on malaria, its severity, and preventive methods; trust in the strategy, its apparent efficacy to protect against malaria, and ease of use; and happiness to recommend the strategy to others. For healthcare staff, the aim was to gather their opinions and attitudes towards SMC, data on the staff required to administer SMC, the ease of drug administration, and the overall costs of implementation, including logistics, storage, and workload. Data about the first dose of SMC taken were recorded through a standardized form in order to assess children’s adherence to the strategy.

### 2.7. Sampling and Sample Size Calculation

#### 2.7.1. Qualitative Approach

The qualitative sampling approach consisted of purposive sampling. For the selection of participants in the community, maximum variation purposive sampling aimed at selecting key informants likely to have different points of view was favored. The main criterion used was ethnicity (which has a strong influence on health beliefs and habits in this rural area). As for health workers, those likely to play a role in the implementation of the SMC were included in the study. We organized 8 focus groups: 4 focus groups with mothers and 4 with fathers, i.e., 2 focus groups per village (one for mothers and one for fathers). The number of participants was between 8 and 12. The village of Diendron, due to a cultural activity, was unable to participate in the focus group. Twelve health workers were interviewed.

#### 2.7.2. Quantitative Approach

The sample size was determined based on the formula below:

$$n=\frac{Z_{\alpha }\times p\left(1-p\right)}{d^{2}}$$

We assumed a coverage rate for the strategy of around 50% (*p*), with a 95% confidence interval (i.e., *Za* = 1.96) and a 2.5% precision rate (*d*). The expected sample size was adjusted by adding 10% as the probable non-response rate and set the number of children expected to join the study to 1700.

To assess acceptability, a sub-group of 264 caregivers was selected from main sample after the awareness campaign based on Stuart Pocock’s formula below, with *f(α, β)* = 10.5:

$$n=\frac{p\times \left(1-p\right)}{d^{2}}\times f\left(\alpha ,\beta \right)$$

We assumed 50% acceptability, with a 10% precision rate.

The following approach was adopted to assess acceptability and feasibility according to the proportion of doses received by children during the coverage survey carried out one month after the 5th cycle of SMC. SMC consisted of the administration of three daily doses of sulfadoxine–pyrimethamine for a month in such a way that first dose of amodiaquine and the unique dose of sulfadoxine–pyrimethamine were administered under direct observation of the community field workers (in charge of the strategy delivery), and then the remaining day 1 and day 2 amodiaquine doses were released to the mothers with clear advice on how to administer them.

We assessed the acceptability through the proportion of daily doses given to the enrolled children by the mothers and those who effectively administered the remaining doses to their children. We used in this study the proportion of children who were administered second and third doses as an indicator of the mothers’ adherence to the strategy. We used the Schwartz formulae to reach adequate sample size to assess the adherence to the doses administered at home by the mothers, where *Zα* = 1.96 for 80% power and a 95% confidence interval, *p* = 70% proportion of mothers who adhered to the indication to administer the second and third doses at home, *e* = 5% degree of precision, and *c* = factor of correction for the clustering effect. Thus, the expected sample size was 484 mothers; adjusting for 10% non-respondents yielded a final sample size of 532 mothers. Participants were randomly selected to complete the survey. The random sampling method was used to enroll participants.

### 2.8. Data Management

For the qualitative study, interviews were recorded, then transferred to a computer for transcription. Thematic analysis was performed using NVIVO 11.0 software: Open coding was used in order to leave plenty of room for discovery. We carried out an initial coding based on the relevant words that emerged from the comments of the stakeholders surveyed. Nodes were obtained using a query to identify the most frequent and relevant words and expressions. As Christophe Le Jeune (2014) suggests, this involved paying attention to the actors’ experiences, feelings, emotions, opinions, and representations, over and above the thematic structure of the interview guides used. We created these nodes by going through the verbatims to extract the meaning that the actors attributed to their lived experiences. The data were sorted using the different nodes obtained on the basis of these two coding operations.

For the quantitative approach, data were collected onto tablets where the case report form was already pre-loaded. The adherence data were collected on the device using ODK software version v2021.2.4 on a daily basis, then transferred to a local server for checking, reconciliation, and cleaning purposes. Clean data were then retransferred to the server for analysis purposes. SPSS version 17.0 was used for the data analysis. Descriptive statistics were computed, as well as univariate and multivariate analysis. A significance level of 5% was considered statistically significant.

### 2.9. Ethical Considerations

The study protocol was submitted to the National Ethics Committee for Life and Health Sciences of Côte d’Ivoire for approval prior to any fieldwork. Meetings were held with local communities to explain all aspects of the study (procedures, risks, benefits). During these meetings, it was made clear that participation in the study was free and that there were no constraints for those who were not willing to be enrolled (this would not affect their care seeking). No payment was made to the study participants. However, any prescribed treatment was provided free of charge to the participants. All informed consent documents are kept in regulatory files (locked up). The data collected in this study are anonymized as far as possible, so the risk of loss of confidentiality is very low. Access to the data is limited to team members directly involved in the data acquisition and to members of the ethics committee or the clinical monitor, who may want to check the compliance of the conduct with the approved protocol.

## 3. Results

### 3.1. Baseline Characteristics

A total of 1701 children eligible to receive SMC were enrolled and followed up with during all five SMC cycles from July to December 2021. Dikodougou, which was the biggest and main site, had most children enrolled. Table 1 shows the distribution of children across the study sites.

### 3.2. SMC Acceptability Assessment 

SMC acceptability was assessed based on the opinions of health staff and families through the mothers during the coverage survey.

Health staff: Overall, of 14 health staff who were invited for the individual interviews, one nurse and one field worker declined the invite to participate; 12 effectively attended. The profile and number of attendee were five community health workers selected from among the distributors of chemoprevention drugs to children, two nurses supervising the activities of the community health workers, the head doctor of the district hospital where the strategy was implemented, the pharmacist managing the drugs, the health action manager responsible for coordinating chemoprevention, the head in charge of epidemiological surveillance in the health district, and the departmental director. We noted that all respondents were happy with and favorable to the implementation of the seasonal malaria chemoprevention, as witnessed by their declarations.

### 3.3. From the Health Staff

Health staff members gave a favorable opinion about SMC after seeing the malaria trend go down since the children started taking the drugs. According to their statements, SMC reduced malaria morbidity in the routine in the catchment area of all sites involved in the study since the strategy was rolled out.

The first health staff declared, “*Before this project happened, there were children every two weeks or every month, the parents were forced to look for money to seek care for their children at the hospital. My own friend was in this case...every month someone came to wake me up at night to send his child to the hospital so I have to inform the senior nurse that somebody is coming to the health facility, even if it’s 3:00 a.m. But since we started using these medications (SMC), we have no longer sent him to the hospital and the last time was 4 months ago*”.

The second health staff added, “*Indeed, the medications are good for prevention: from the beginning until this 4th visit, the children did not get sick much. We noticed this even in hospitals, the observation was made at the hospital level. Children come to the hospital less frequently*”.

Health workers have supported SMC because it has demonstrated the capability to reduce malaria-related morbidity in children under 5 years old. In addition, they believed that SMC could lead to better results if associated with other prevention tools:

“*I think this project is very welcome: if we combine it with other prevention strategies, it will be even more effective. With SMC, I think we can really reduce the malaria mortality rate*”.

The CHWs interviewed wanted the project to continue and be extended, as shown by this statement:

“*The first thing I’d like is for the SMC to be extended to the whole population and for medicines to be given to everyone. All the villages should benefit from this prevention because it’s effective. Now we ourselves, at our level, are ready to help the population, because malaria makes people tired. If we’re given the means, we’re ready to work for the SMC because it’s good for the children and all*”.

Health workers also testified that parents were happy with SMC. They received feedback from them showing that SMC reduced the occurrence of malaria:

“*At the SMC, in any case, many mothers have confirmed since the third cycle that during this three-month period, they had not visited the hospital for a case of malaria in their children*”.

“*Well, with the feedback, the visits to households, some households have said that since they started taking the drugs, their children no longer have malaria as they did before, so I think that this chemoprevention is effective*”.

The acceptability of SMC can be summed up by this statement from a health official:

“*The chemoprevention distribution activities went very well, although there was some initial reluctance due to unfamiliarity with the drugs. Communication with the people concerned removed this reluctance, and the population was in favor of taking SMC. This strategy would be welcome again next year*”.

### 3.4. From Mothers/Guardians and Families

In the quantitative survey, parents in Dikodougou were asked four questions to assess their acceptance of the strategy: their trust in the strategy to prevent malaria, the ease of drug use, the ability to adopt SMC as a malaria prevention measure, and finally, the likelihood of recommending SMC to a parent. For the responses, 97.8% (272/278) of interviewed mothers declared that they trust the SMC to prevent their children from getting sick (with malaria). In addition, 94.6% (263/278) indicated that the strategy was easy to implement or that it was easy to administer the drug at home, 98.9% (275/278) were prompt in adopting SMC as a preventive strategy against malaria. Globally, 97.8% (272/278) were ready and happy to recommend SMC to a friend or parent. The focus group discussions with parents confirm the qualitative data. One mother even stated:

“…*before the medicines arrived, the day before I went to the hospital with my child. Now when the medicines arrived that I started using, I really didn’t go to the hospital anymore until now (4th visit). The medicine is very effective*”. Another mother confirmed the effectiveness of SMC by comparing children who received chemoprevention with those who did not: “*We really felt a change in those who took the medication (preventive treatment). They didn’t go to hospital for malaria. But those who haven’t taken it are still going to hospital with malaria. So, the medicine is good and it does us good*”. Another mother declared, “*I find the medication very effective, as they haven’t been to hospital since my children started taking it. This time last year, we were going to hospital all the time, and I’m happy about that*”.

As far as the drug safety is concerned, the drugs were overall well tolerated, as stated by mothers following the drug uptake:

“*No, my two children who are taking chemoprevention had no problems at all after taking the drugs; they were just emboldened; it’s nothing to do with the drugs—on the contrary, I’m happy because SMC is a good thing*”.

“*No, no, there were no problems with the children who took the medicines in the village; only one mother said that her child was very tired after taking the medicines. My child and the other children in the village were fine. I even think that the medicine improved their health”. Only one mother who reported that her child had asthenia following the drug administration but this transient and did not require the interruption of the treatment. Fathers’ comments were similar to those of the mothers. But what was specific to their attitude was their wish that SMC be extended to all children and even to adults: “Since my three children started taking this medicine, they have not yet fallen ill. It’s the older ones who don’t take it who have recently come down with malaria. I hope that all the children, even the older ones, will take the medicine. We have to give the medicine to the whole village because malaria is a nasty disease*”.

“*You see, I have a child who was accompanied to the hospital all the time because of malaria, last year he was even hospitalized… But when the medicine was distributed, I myself kept an eye on his mother so that he would take it well. You see, in the first month, he had malaria so we didn’t give him the medication. Since the second month he has been in the program and so far, he has no more malaria. Such treatments must benefit the entire population, because here we are very tired of malaria”. “SMC’s drugs are so good that everyone has to take it to kill the malaria that is already in our bodies*”.

Nevertheless, among fathers, two were less enthusiastic about SMC. For one, as the strategy was new, he needed more time to form an opinion: *“Well, the medicines are new, we’re told that they can’t harm the children and there’s even a doctor who often comes to the village to check that the medicines aren’t tiring the children… the parents say that the medicines are good, but I’m still waiting to see”.*

For the other, there were already effective means of prevention—in particular, environmental sanitation, which he thought should be promoted: “*I think there are too many methods of prevention at the same time. Maybe we don’t need all that, if people clean their homes and front yards properly too, mosquitoes will disappear and malaria with them*”.

In addition to the assessments mentioned previously, we directly interviewed 536 mothers with children who may be eligible for SMC in order to assess the completeness of all doses, which might be an indicator of their acceptability. We questioned mothers about taking second and third doses of SMC during the coverage study. SMC is commonly implemented by letting community health workers observe the first dose and having the mothers be in charge of the second and third doses. The mothers were asked to bring the blister used during the fifth cycle and confirm if all doses were missing inside. We noticed the proportion of mothers who gave the second and third dose increased over the cycles to reach 92.3% (286/310) adherence during the last cycles of SMC.

### 3.5. Feasibility 

**Figure 1:**
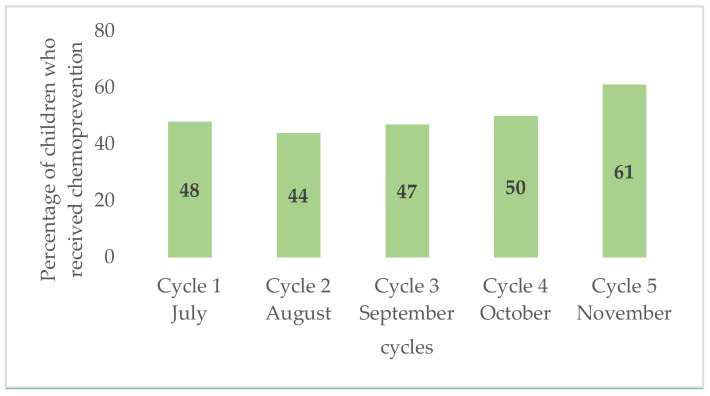
Comparison of SMC coverage by cycle.

This parameter was assessed considering three key factors: availability of trained staff, affordable good-quality drugs, logistics, time needed, and additional workload for the health system. Upon request, qualified staff for the work were available, who received a short training on the procedures and techniques along with a modest incentive. In concrete terms, to run the campaign for 1701 children over 5 months, corresponding to five SMC cycles, eight CHWs were mobilized to distribute the medicines, two nurses to supervise the work of the CHWs, one doctor to monitor adverse reactions, one head of the health action department as the local coordinator of the campaign, and one pharmacist to manage the medicines. We used the door-to-door strategy to deliver SMC (already used in SMC settings), and this context, it was feasible. The teams reached 90–100% of families to deliver drugs over the full 5-month period. The time taken to administer the drug was minimal (15–20 min), thus increasing the acceptability. As SMC is largely recommended, the availability of the drugs has significantly improved. The procurement of the drug from the manufacturers was easy and no delay was observed in the drug delivery. Figure 1 shows SMC coverage by cycle, which is a key point in the implementation of SMC. There was a steady increase in coverage over the cycles.

## 4. Discussion

We evaluated the acceptability and feasibility of five cycles of SMC in the Dikodougou health district. Following the latest updated WHO guidelines, the northern part of Côte d’Ivoire is eligible for SMC [2]. Overall, the strategy in this study was well accepted and feasible. From the health workers’ perspective, SMC is welcome; it reduces the frequency of sickness episodes (malaria) so children are less in need of care, with the consequence being that health facility staff are less solicited by sick children’s parents. Despite sustained effort and a reduction in the incidence ranging from 5964 episodes for 1000 person-years in 2019 to 44. 097 episodes for 1000 person-years in 2020, the burden of malaria remains high [7]. 

The acceptability of SMC could lead to a reduction in malaria burden for the health system. The mothers were happy with the SMC strategy, as after the drug administration started, their children experienced less malaria compared to those who did not receive SMC. The perceived efficacy of SMC in keeping the children free of malaria sustained their acceptance of the strategy. These findings are in line with an acceptability and feasibility study conducted in Ghana among mothers and care givers. This study reported a positive opinion from the mothers about SMC, as they recognized the strategy’s benefit in protecting their children against episode of malaria [8]. In Burkina Faso, SMC has been implemented in four cycles since its introduction. Recently, the acceptability and feasibility of the extension to five cycles were assessed in the health district of Mangodara. Overall, adding a fifth cycle was perceived as acceptable by caregivers, community distributors, and stakeholders due to the positive impact on the health of children under five [9]. However, the largely positive opinion reported in these studies is accompanied by remarks that could potentially affect the acceptability and feasibility. Of these, the period of the strategy implementation played a major role. Indeed, the peak transmission period coincides with the peak of labor in the field. Most parents are displaced in these remote areas that are hard to reach, preventing the availability of several contacts to receive the daily doses of SMC over consecutive months [9]. Furthermore, physical access to SMC medication, the drug regimen, trust in the medical profession, and perceived norms around malaria prevention all likely influenced caregivers’ level of uptake [8]. Adherence to monthly cycles and daily doses is an objective parameter that can estimate the strategy’s acceptability; the coverage of the intervention has increased over subsequent cycles, reaching nine out of ten children. Similar high coverage has been reported in countries already implementing SMC, such as Burkina Faso, Niger, and Mali [10,11,12]. Few transient minor/middle adverse events were reported but did not require treatment interruption and therefore did not impact the adherence and thus the acceptability of the strategy.

## 5. Conclusions

This study’s results show that most participants were happy with SMC and looked comfortable with this strategy. That might be a good indicator showing that SMC is acceptable and feasible in the northern part of Côte d’Ivoire. However, if acceptability and feasibility are key points in large-scale SMC implementation, only high coverage of eligible children and doses completeness can make this strategy work better and reduce malaria prevalence in a routine manner. Further studies may be needed to explore challenges about reaching and maintaining a high coverage of SMC in the community.

## Figures and Tables

**Table 1:** Summary of enrollment by site.

Sites	Dikodougou	Kadioha	Poundia	Sonzorisso	Diendron	Total
Children enrolled	1146 (67.4%)	312 (18.3%)	129 (7.6%)	93 (5.5%)	21 (1.2%)	1701

## Data Availability

The data presented in this study are available on request from the corresponding author.
